# Supplementary figures and images for: Autoimmunity-associated allele of tyrosine phosphatase gene PTPN22 enhances anti-viral immunity
Source: PLoS Pathog. 2024 Mar 21;20(3):e1012095. doi: 10.1371/journal.ppat.1012095 (PMC10987006; doi:10.1371/journal.ppat.1012095)

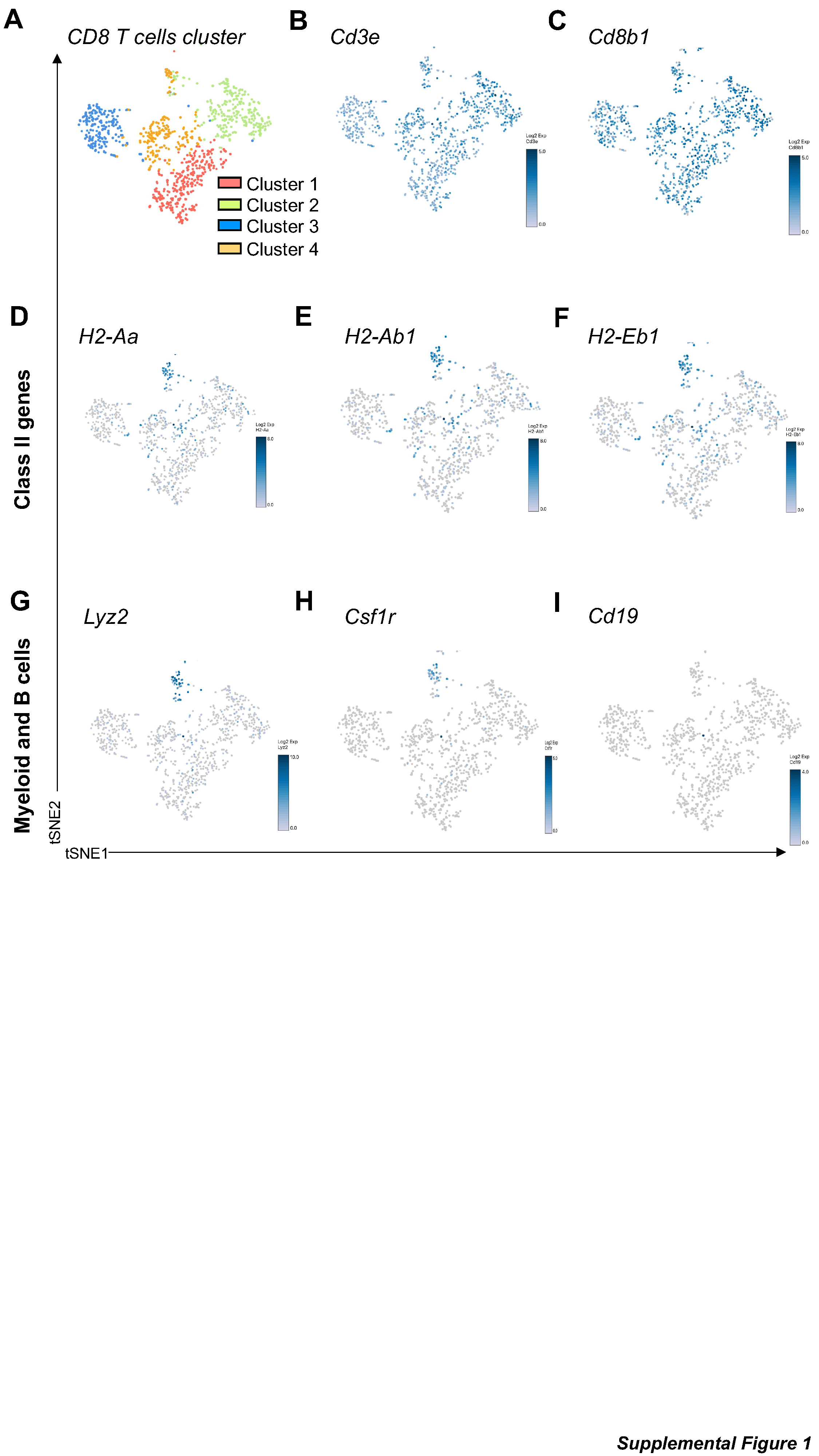

Supplement: S1 Fig — CD8 T cells were reclustered within Loupe software using aggregated file containing both PEP-WT and PEP-619WW data. tSNE visualizing Clusters 1–4 of CD8 T cell re-cluster analysis. Cluster 1 salmon, Cluster 2 light green, cluster 3 blue, and Cluster 4 light orange (A). Average gene expression of genes Cd3e (B), Cd8b1 (C), H2-Aa (D), H2-Ab1 (E), H2-Eb1 (F), Lyz2 (G), Csfr1 (H) and Cd19 (I). Gene expression shown in Log2 scale, range is on heat map scale for each tSNE plot. (TIFF) [file ppat.1012095.s001.tiff]

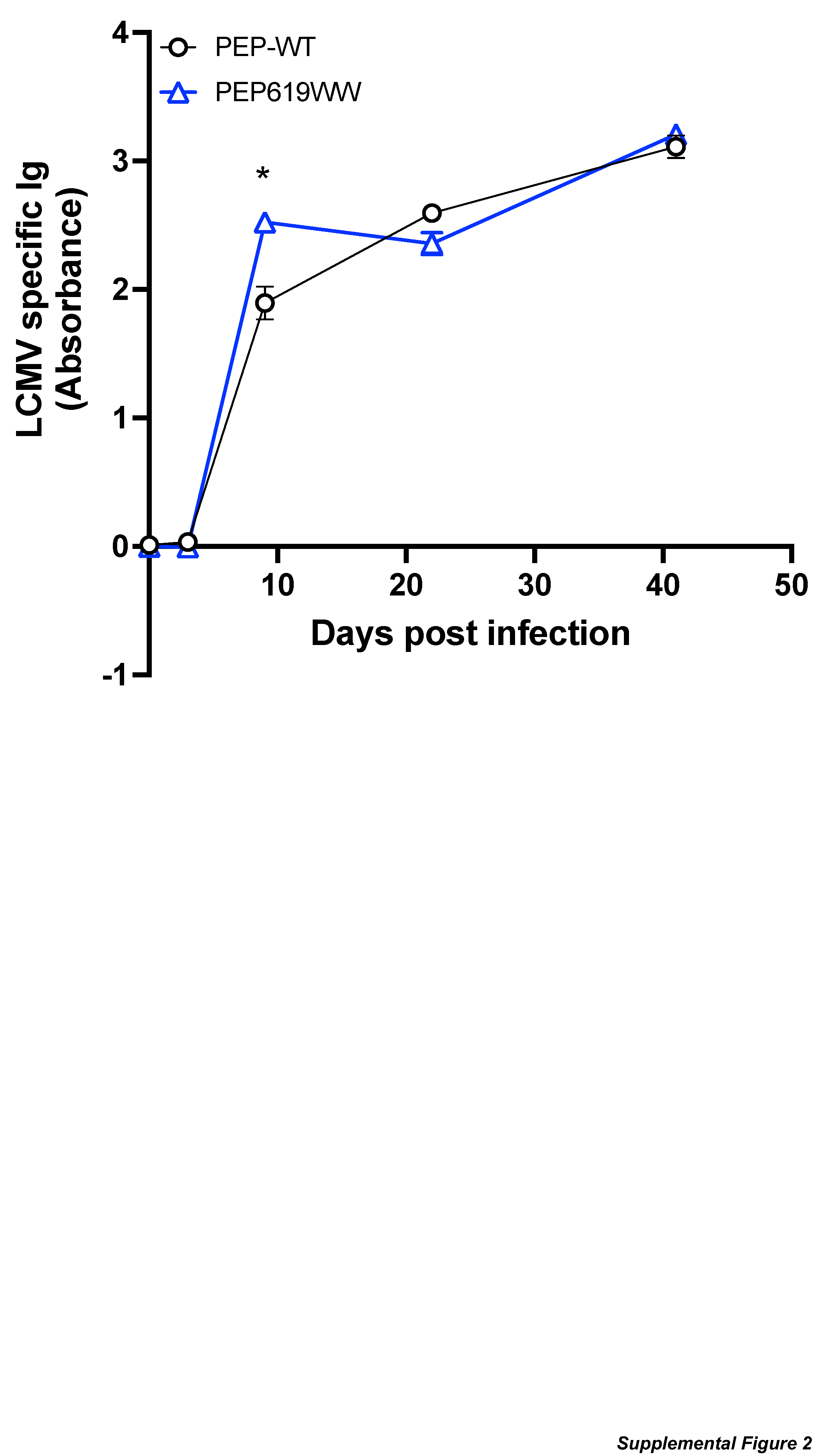

Supplement: S2 Fig — Serum from infected PEP-WT (black) and PEP-619WW (blue) taken at 0, 3, 9, 21, and 41 days post infection and measured for neutralizing IgG2A antibodies. Data shown from a 1: 1583 dilution. Representative experiment shown. SEM shown. *p<0.05; T-test at each time point between genotypes. (TIFF) [file ppat.1012095.s002.tiff]

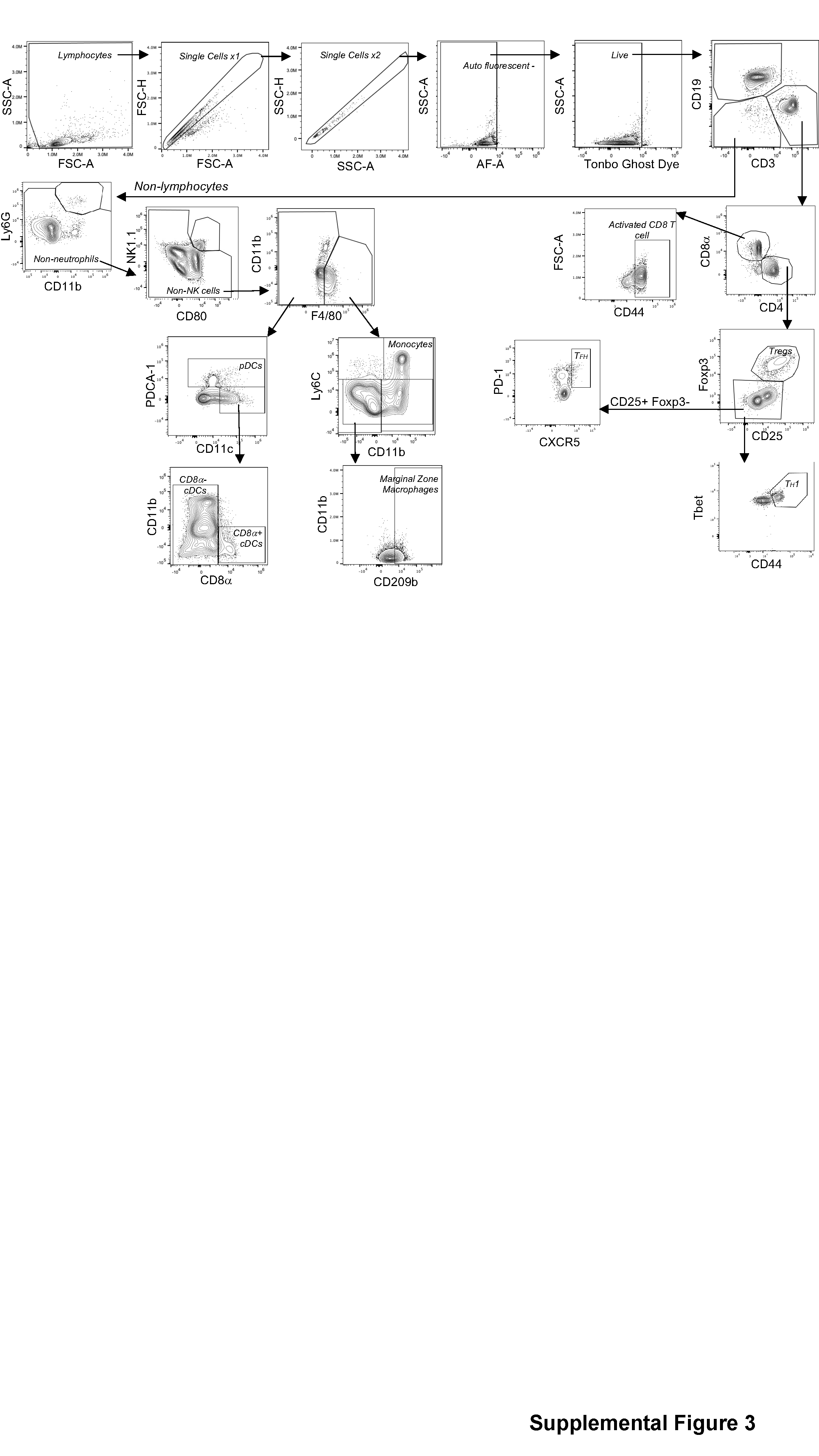

Supplement: S3 Fig — Representative gating strategies to identify populations of interest presented throughout this manuscript. Populations are labeled on flow cytometry plots. Arrows indicated down gating of the population where they are drawn from. Gating strategies are also listed in figurelegends where the data appears. (TIFF) [file ppat.1012095.s003.tiff]
